# Editorial: Digital Health for Palliative Care

**DOI:** 10.3389/fdgth.2022.888419

**Published:** 2022-04-07

**Authors:** Cathy Payne, Haridimos Kondylakis, Lefteris Koumakis

**Affiliations:** ^1^European Association for Palliative Care, Vilvoorde, Belgium; ^2^Institute of Computer Science, Foundation for Research and Technology (FORTH), Heraklion, Greece

**Keywords:** palliative care, MHealth (mobile health), health informatics and information systems, policy recommendations and future areas of research, decision support system

Palliative care has recently been determined as a population health need and an integral part of universal healthcare delivery (1). The World Health Organization defines palliative care as “an approach that improves the quality of life of patients and their families facing the problem associated with life-threatening illness, through the prevention and relief of suffering by means of early identification and impeccable assessment and treatment of pain and other problems, physical, psychosocial and spiritual” [(2), p. 94]. Whilst a medical specialty in its own right, awareness and training in the principles and practices of palliative care is a requirement for all health and social care professionals and support staff. In 2022, EAPC revised their recommendations on standards and norms for palliative care in Europe (3). These recognize the importance of technological advances in palliative care, such as the widespread use of digital health records, to support better information exchange.

In this special edition of Frontier in Digital health, we were particularly interested in highlighting novel digital solutions that could (i) improve quality, effectiveness and cost-effectiveness of palliative care services as well as access to care, (ii) reduce symptom burden and suffering (iii) improve well-being of patients in need of palliative care and their formal and informal caregivers, (iv) reduce economic and wider societal burden arising from increased numbers of patients in need of palliative care, (v) improve clinical guidelines and policy recommendations with respect to pain management, palliative care of patients with life-threatening diseases.

The papers in this edition are broad in their scope, ranging from early piloting of smart technologies through to exploring the ethical principles and process fidelity behind digital intervention research^1^.

The special issue begins with a **systematic review** on the economic evaluation of digital health interventions in palliative care (Naoum et al.). More specifically the authors showed that research on digital health interventions is mostly focused on symptom management with the potential to be as cost-saving and cost-effective as usual care. However, the generalizability and the wider applicability of the evidence is unclear due to the scarcity of research, showing that more research is required in the area.

Two of the presented articles focus on **hematologic diseases and cancer**. More specifically the use of a patient's self-assessed health status has become a valuable tool in clinical practice for chronic diseases and lately a number of digital interventions aiming to support palliative cancer care have been proposed. Koumakis et al. introduce a digital platform for palliative care that includes a mobile app for patients and a tool for healthcare professionals offering a unique user experience to patients, doctors/researchers and caregivers. Authors followed a user centered process for development, while the platform is in the phase of pilot testing to evaluate its feasibility and its potential impact on the quality of life of palliative care patients with hematologic malignancies. AquaScouts (Hoffmann et al.) on the other hand, is a serious game designed for supporting palliative care for children with cancer. The authors provide insights on the participatory design required for such a serious game, the validation process and also demonstrate a number of challenges that should be overcome when deploying eHealth interventions to support care delivery.

**Wearables** can also play a key role in monitoring and assessment of symptoms of advanced diseases. In their paper, Mallick et al. consider how clinicians might remotely monitor symptoms of breathlessness (dyspnoea) through the use of a smart patch. Whilst still in the early stages of development, the study demonstrated the potential that wearable health technology has in assisting healthcare professionals in the assessment and management of symptoms of advanced disease without adding an additional burden of patient symptom reporting.

**Machine learning** can also be useful for helping clinicians with their daily tasks. Haas et al. propose an interpretable machine learning model based on association rules mining for the prediction of anxiety in palliative patients. The algorithm takes into account 40 demographics and medical variables from routine data in order to generate the decision rules. Results of the evaluation with the hospice and palliative evaluation (HOPE) dataset are promising and indicate that such a model could be used in clinical practice for earlier detection of anxiety.

Recognizing and responding to the **challenges and pitfalls** for implementing digital health solutions in clinical studies is the focus of our final papers in this special edition. In Meyerheim et al., the authors present recommendations on avoiding delays and overcoming the barriers in the planning of prospective studies for palliative care interventions. More specifically they show that good preparatory work can pave the way into an increased efficiency of the planned interventions lifting ethical, legal, administrative and clinical barriers. Garani-Papadatos et al. focus more specifically, on the ethical ramifications of digital intervention technologies for palliative care, addressing the normative challenges arising including (i) privacy (ii) data protection and transparency, (iii) fairness, (iv) individual wellbeing (v) acceptability and propose that ethical evaluation methodological frameworks.

## Conclusions

We need a culture in palliative care that not only values technological advances and the idea of research to support the use of technology as an aid to palliative care interventions, but that actively builds “research capital” by supporting partnerships between those working in the specialist fields of palliative care, computing, big data modeling, engineering etc. We hope that this special issue goes some way to supporting this endeavor by showing how working across boundaries has value for all those working within this emerging field.

## Author Contributions

All authors listed have made a substantial, direct, and intellectual contribution to the work and approved it for publication.

## Funding

The research was supported by the MyPal project which has received funding from the European Union's Horizon 2020 research and innovation program under the grant agreement No. 825872. This article reflects only the author's view.

## Conflict of Interest

CP was employed by European Association for Palliative Care. The remaining authors declare that the research was conducted in the absence of any commercial or financial relationships that could be construed as a potential conflict of interest.

## Publisher's Note

All claims expressed in this article are solely those of the authors and do not necessarily represent those of their affiliated organizations, or those of the publisher, the editors and the reviewers. Any product that may be evaluated in this article, or claim that may be made by its manufacturer, is not guaranteed or endorsed by the publisher.

## Footnotes

^1^ https://www.frontiersin.org/research-topics/17436/digital-health-for-palliative-care
